# Effect of genotype and genotype by environment interaction on total cyanide content, fresh root, and starch yield in farmer‐preferred cassava landraces in Tanzania

**DOI:** 10.1002/fsn3.345

**Published:** 2016-02-09

**Authors:** Mariam K. Mtunguja, Henry S. Laswai, Edward Kanju, Joseph Ndunguru, Yasinta C. Muzanila

**Affiliations:** ^1^ Mikocheni Agricultural Research InstituteP.O Box 2662Dar es SalaamTanzania; ^2^ Department of Food Science and TechnologyFaculty of Agriculture, Sokoine University of AgricultureP.O Box 3006 Chuo Kikuu, MorogoroTanzania; ^3^ International Institute of Tropical Agriculture (IITA)P.O Box 34441Dar es SalaamTanzania; ^4^ Department of Biological SciencesFaculty of ScienceSokoine University of AgricultureP.O Box 3038MorogoroTanzania

**Keywords:** Cyanogens, GGE biplots, *Manihot esculenta*, maturity period, starch yield

## Abstract

High starch yield is the most important trait for commercialized cassava starch production. Furthermore, cyanide present in cassava roots poses a health challenge in the use of cassava for food. Cassava genotypes have varying maturity periods that are also environmental dependent. This study aimed at identifying suitable cultivars and optimum time of harvest to maximize starch production across three environments. The study found significant difference between genotypes, locations, harvest period, and all the interactions (*P *≤ 0.001) for all traits analyzed. Kiroba recorded high starch yields of 17.4, 12.7, and 8.2 t ha^−1^ at Chambezi, Amani, and Magadu, respectively. Kilusungu recorded highest cyanide content of 300–400 ppm across all locations but Kiroba recorded highest values of 800 ppm, 15 months after planting at Chambezi. Genotype by environment (GGE) biplot analysis revealed that Kiroba was a superior cultivar in terms of starch yield. Kilusungu recorded highest cyanide content and average starch yield, therefore suitable for use in starch production. The study confirmed effect of genotype and genotype by environment interaction, Kiroba cultivar was superior in terms of starch yield and maximum starch yield was obtained at 9 months after planting. Nyamkagile and Kibandameno had the lowest cyanide content across all environments.

## Introduction

Starchy tuberous roots of cassava provide food for people in sub‐Saharan Africa and Latin America (Nassar and Ortiz 2007). Apart from culinary requirements, cassava starch can also find application in various industries such as pharmaceuticals, cosmetics, biopolymers, textile, and biofuels (Nassar and Ortiz 2007). Cassava productivity (8.5 t/ha fresh weight) in Tanzania is low (Mkamilo and Jeremiah 2005) compared to average world potential of 35 t/ha (Lebot 2009), due to various production constrains (El‐Sharkawy 2004; Siritunga and Sayre 2004).

The increasing demand of starch for Tanzanian industries, which in 2011 was estimated at 5781 tons per year, was equivalent to 3.2 million US dollar (Tanzania Revenue Authority (TRA) 2011). This demand can be met by cassava farmers and the potential to fulfill the demand is available. Cassava farmers can benefit from marketing cassava to starch producers thereby improving their income and food security (Tonukari 2004). To achieve this, adequate information is required on the right cassava cultivars and appropriate harvesting period so as to maximize cassava production and increase income. Maximum yield of cassava is attained after full development of the canopy, beyond which the root growth decreases (Sagrilo et al. 2006). Cassava has varying maturity period depending on genotype (Ceballos et al. 2004). Moreover, studies have reported that definite optimal harvest time for cassava is genotype and environment dependent (Benesi et al. 2008). There is a need to maximize cassava productivity by selection of genotypes with high dry root and starch yield.

Santisopasri et al. (2001) working on six Thailand cassava cultivars revealed that environmental conditions during both initial and late growth stages are necessary for satisfactory root growth and starch quality. Water stress in early development stages of cassava affects its productivity (Santisopasri et al. 2001; El‐Sharkawy 2006). Also insufficient maturity impairs starch quantity and quality (Santisopasri et al. 2001). Furthermore, high productivity of cassava will not be achieved until the right environmental conditions have been met (Benesi et al. 2008; Egesi et al., 2007). Therefore, appropriate locations for growing cassava cultivars should be identified precisely.

Cyanide is produced by plants as a by‐product of ethylene metabolism, or as reduced form of nitrogen storage and defense against attack by herbivorous (Bennett et al.*,* 1994; Møller 2010). Cassava plants produce high quantities of cyanogenic compounds compared to other crops, and it is mainly concentrated in leaves and roots. The cyanogenic potential varies with genotype (Burns et al. 2012) and within the same genotype; cyanide is also affected by planting season and soil type (Bokanga et al. 1994). Thus same genotype can taste sweet in one locality and bitter in another (Bokanga et al. 1994; Bradbury et al. 2013). Konzo is the irreversible upper motor neuron damage caused by consumption of high dietary cyanogens especially from insufficiently processed cassava roots. It occurs to children and women of child‐bearing age. Also, high dietary cyanogens have been reported to cause Tropical Ataxic Neuropathy (TAN) in elderly and stunted growth to children ((Nhassico et al. 2008). During food shortage, short cuts in processing cause Konzo outbreak (Siritunga and Sayre 2004; Mlingi et al. 2011).

Farming communities where cassava is a staple food, prefers high cyanogenic (bitter) cultivars as protection from predators and thieves (Mkumbira et al. 2003; Siritunga and Sayre 2004; Benesi et al. 2010). Bitter varieties are preferred for their high yield (Bradbury et al. 2013) and production of flour with characteristic taste (Chiwona‐Karltun et al. 2004; Dufour, 1994). Bitter varieties have also long durability thus can be stored underground and harvested piecemeal (Mkumbira et al. 2003; Chiwona‐Karltun et al. 2004. As a result, food insecure farmers prefer growing bitter cassava. Commercial starch production will increase demand of cassava in the market and reduce dependency on bitter varieties as food reserve, thus decreasing chances of cyanide exposure and associated risks.

Only few local cassava cultivars have been evaluated for dry matter and in less diverse environmental conditions (Mtunda 2009). Also, cyanogenic potential of commonly grown Tanzanian cassava cultivars has not been established. In this study, six farmer‐preferred cassava landraces were evaluated in diverse environment in terms of temperature range, rainfalls distribution, and soil conditions. These landraces have long survived biotic and abiotic stresses, and are preferred by farmers. These characteristics make them ideal candidates for recommendation for commercial production of both food and starch. Therefore, this study aimed to determine the effect of genotype and environmental conditions on starch yield, cyanogenic potential across sites, and harvesting periods, and finally to evaluate stability and superiority.

## Materials and Methods

### Cassava landraces and trial design

A total of six farmer‐preferred cassava landraces collected from Eastern zone of Tanzania in January 2012 were used for this study. The landraces included Nyamkagile, Kibandameno, Kilusungu, Msenene, Kalolo, and Kiroba. A split‐plot design replicated three times using plot size of 5 m × 5 m with spacing of 1 m × 1 m was deployed, whereby the landraces were main plots and harvest round as subplots. Trials were planted during the long rainy season, and harvesting took place in three rounds, namely 9, 12, and 15 months after planting (MAP). Three healthy plants were uprooted from each plot, marketable roots were selected, counted and weighed (SRM). For starch and dry matter analysis, roots were brought immediately to the laboratory of Food Science and Technology, Sokoine University of Agriculture (SUA).

### Location of trial sites and edaphic conditions

Trials were set at Chambezi (Bagamoyo), Amani (Muheza), and Magadu (Morogoro municipality) from April 2012 up to July 2013 (Table 1). Weather data for minimum and maximum temperature and monthly total rainfall of trial sites were recorded. Soil samples were collected from the top 30 cm at different three positions for each location (Gullberg et al. 2007). Soil samples were kept in bags and transported to the Soil Science laboratory of Sokoine University of Agriculture for analysis following methods by Soil Science Society of America (SSSA) (1996)

**Table 1:** Description of location for the study

Location of the trial	GPS coordinates	Agro‐ecological zone	Altitude (m)	Temperature Range (°C)		Annual rainfall (mm)
				Min	Max	
Chambezi‐ Bagamoyo	S 06.55318E 38.9148	Coastal plains	46	19–23	29–31	800–1000
Amani‐ Muheza	S 5.1088E 38.67373	Eastern plateau and mountain block	542	15–18	27–30	800–1500
Magadu‐Morogoro	S 6.84706E37.65448	Eastern plateau and mountain block	1100	19–23	29–31	600–1000

### Total fresh root yield (FSRY)

FSRY (t/ha) was calculated from the formula:

FSRY=(SRM×10,000)/(3×1000)

SRM = Storage Root Mass.

### Root dry matter content

Cassava root dry matter (DM) was determined according to Benesi et al. (2008). Fresh cassava root were grated and exactly 200 g of the grated cassava root (W_1_) were put in preweighed petri dishes (W_0_). The samples were oven dried at 60°C for 72 h, and weighed (W_2_) after removal from the oven. DM was calculated as follows;

$$\text{DM}\left(g/\text{kg}\right)=\frac{W_{2}-W_{0}}{W_{1}-W_{0}}\times 1000$$

### Starch content (SC) expressed as fresh weight of cassava

Starch extraction was conducted following the method by Benesi et al. (2010) with slight modification**.** Exactly 500 g of fresh peeled cassava from three root samples were chopped using water as extraction solvent in a laboratory blender. The tissues were broken and fiber separated by sieving through double layer of cheese cloth. After washing several times, starch was oven dried at 50–55°C for 72 h and then weighed (Wo).

SC (g/kg) was calculated as = Wo/500*1000

### Total starch yield

Starch yield was obtained as the product of fresh cassava yield and starch content expressed as t ha^−1.^

Starch yield = SC*FSRY.

### Cyanide content

Cyanide content was determined following the picrate paper method as illustrated by Bradbury (2009). Root sampling was standardized to account for known root variation in cyanide concentration and analysis was done within an hour after harvesting. Standardization was done with standard paper and a blank provided with the kit.

Ppm = mg HCN equivalents/kg fresh root.

### Data analysis

Data analysis was done using GENSTAT 11 program and Analysis of Variance (ANOVA) for all the traits was performed. When the effects of genotype by environment interactions were significant, further analysis was done using genotype and genotype by environment (GGE) biplot procedure on starch yield and cyanide content. Genotype by environment biplots analysis was performed to determine landrace's superiority, stability, and also which‐won‐where in relation to starch yield and cyanide content (Yan and Tinker 2006).

## Results

### Weather and soil condition of the trial sites

**Figure 1:**
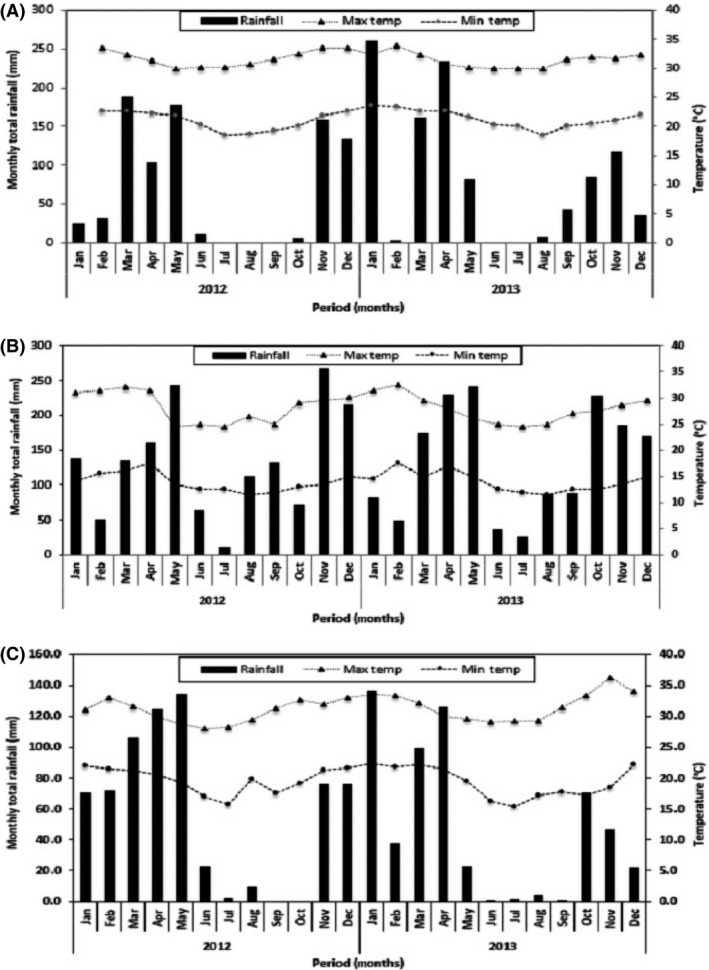
Temperature and rainfall data for the three trial sites (A) Chambezi (B) Amani and (C) Magadu taken from January 2012 to December 2013.

Total annual rainfall was 827 mm, 1605.3 mm, and 694.4 mm in 2012 and 1024.3 mm, 1599.3 mm, and 570.6 mm in 2013 for Chambezi, Amani, and Magadu, respectively (Fig. 1). Amani had a well spread rainfall throughout the year, followed by Magadu. Mean monthly maximum temperature range for the growing period were 29.9–33.8°C for Chambezi, 24.5–32.5°C for Amani, and 28–36.2°C at Magadu. Minimum monthly temperature range was 18.5–23.7°C for Chambezi, 11.5–17.5°C for Amani, and 15.4–22.4°C for Magadu (Fig. 1). Both sites had slight acidic soils, but Magadu soil was the most acidic. Chambezi had sand soils, Amani had sandy, clay/loamy soils while Magadu had sand/clay. Cation Exchange Capacity (CEC) measures the ability of soil to hold essential nutrients and its availability to plants; it was high at Amani (16.2 cmol/kg) and Chambezi had the lowest (8.2 cmol/kg). The Chambezi soil had high iron (Fe) (41.6 mg/kg) content but low in organic matter (0.3%), while Magadu had low Fe (1.556 mg/kg) and high organic matter (1.6%) content (Table 2).

**Table 2:**
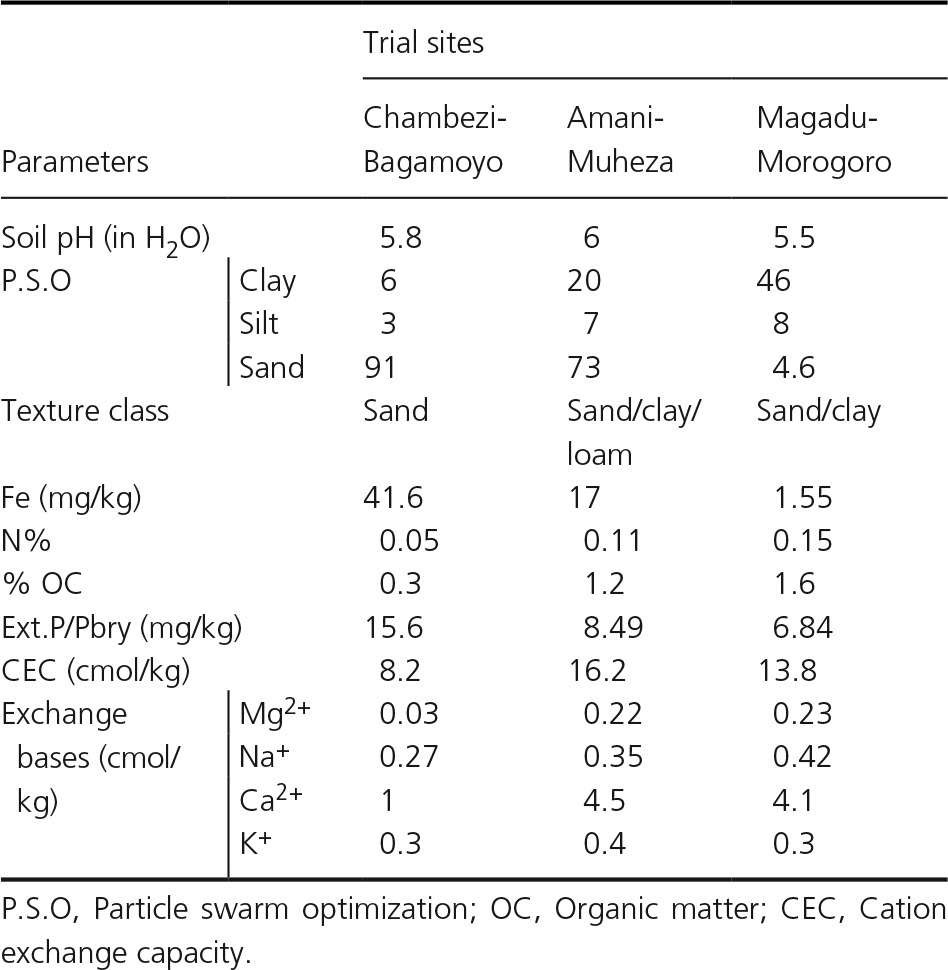
Soil data from the trial sites

### Effect of harvesting time (MAP) on cassava yield, dry matter, starch and cyanide content

The mean fresh root yield at each site and harvest round are given on Table 3 and it varied significantly with harvest round (*P* ≤ 0.001). At Chambezi, there was a yield increase from 9 to 15 MAP for three landraces (Kiroba, Msenene, and Nyamkagile), with Msenene showing the highest increase. The highest yield (39 t/ha) was recorded at 15 MAP from Msenene. At Amani, there was a yield increase from 9 to 15 MAP for four landraces (Kiroba, Msenene, Kalolo, and Kilusungu). The highest yield (25 t/ha) was recorded at 15 MAP from Kibandameno. At Magadu, there was also a yield increase from 9 to 15 MAP from four landraces (Kiroba, Kalolo, Kibandameno, and Nyamkagile). The highest yield (12.6) was recorded at 15 MAP from Kiroba.

**Table 3:** Fresh root yield (t ha^−1^) of six cassava landraces evaluated in three sites at three different harvesting rounds (MAP)

	CHAMBEZI				AMANI				MAGADU			
Landraces (G)	9 MAP	12 MAP	15 MAP	Mean^a^	9 MAP	12 MAP	15 MAP	Mean^a^	9 MAP	12 MAP	15 MAP	Mean^a^
Kalolo	10.5	8.3	19.4	12.7	2.5	3.4	9.8	5.2	1.6	3.7	8.7	4.7
Kibandameno	7.5	5.0	11.0	7.8	5.6	2.9	24.6	11.0	1.9	7.9	10.0	6.6
Kilusungu	7.3	7.3	17.6	10.7	5.5	7.8	18.3	10.5	3.7	12.4	11.9	9.3
Kiroba	4.5	23.0	24.8	17.4	6.5	13.0	18.7	12.7	2.1	9.9	12.6	8.2
Msenene	6.4	12.4	39.4	19.4	9.0	14.4	13.8	12.4	1.2	7.9	6.4	5.2
Nyamkagile	7.4	8.7	19.4	11.8	5.1	7.1	11.6	7.9	1.6	5.2	9.3	5.3
Mean^b^	7.3	10.8	21.9	13.3	5.7	8.1	16.1	10.0	2.0	7.8	9.8	6.6
CV (%)				7.2				7.8				10.0
LSD for G				1.75^c^				0.88^c^				0.45^c^
LSD for MAP				0.91^c^				0.51^c^				0.50^c^
LSD G*MAP				2.42^c^				1.29^c^				1.07^c^
SE for G				0.78				0.28				0.2
SE for MAP				0.45				0.18				0.24
SE G*MAP				1.19				0.45				0.52

CV, Coefficient of variation; SE, Standard error; LSD, Least significant difference; G, Landraces (genotypes); MAP, Months after planting.

a Mean, mean over three harvest rounds.

b Mean, overall landrace site mean.

c *P *≤ 0.001.

The means for root dry matter content at each site and harvest round are given on Table 4, a significant differences (*P *≤ 0.001) was observed. Increase in DM was evident from 9 MAP to 12 MAP but decrease at 15 MAP, a trend observed at all sites. However, Kilusungu recorded highest DM at 15 MAP at Bagamoyo site. Highest DM was recorded at 15 MAP from Kibandameno at Chambezi and Amani site recorded highest overall average dry matter and starch content (Table 5). Starch yield (Table 6) in most landraces showed an increasing trend, with Chambezi sites with highest average starch yields.

**Table 4:** Root dry matter (DM) content (g/kg) six cassava landraces evaluated in three sites at three different harvesting rounds (MAP)

	CHAMBEZI				AMANI				MAGADU			
Landraces (G)	9 MAP	12 MAP	15 MAP	Mean^a^	9 MAP	12 MAP	15 MAP	Mean^a^	9 MAP	12 MAP	15 MAP	Mean^a^
Kalolo	308.0	350.3	285.1	314.5	371.1	388.9	346.6	368.9	344.3	390.9	335.8	356.8
Kibandameno	395.3	411.3	378.1	394.9	397.4	416.8	373.8	396.0	404.0	446.4	373.3	407.9
Kilusungu	306.4	328.6	311.0	315.3	404.2	438.4	382.3	408.3	362.3	383.6	347.2	364.4
Kiroba	367.4	377.7	335.7	360.3	397.5	405.2	375.8	392.9	356.2	405.4	344.7	368.8
Msenene	334.4	365.3	310.4	336.7	349.9	369.3	298.3	339.1	370.1	382.1	328.5	360.2
Nyamkagile	335.6	354.3	332.8	340.9	414.4	425.6	380.1	406.7	402.7	417.2	388.9	402.9
Mean^b^	341.2	364.6	325.5	343.7	389.1	407.4	359.5	385.3	373.2	404.3	353.0	376.8
CV(%)				1.3				2.5				2.5
LSD for G				5.7^c^				8.1^c^				8.6^c^
LSD for MAP				3.0^c^				3.6^c^				6.8^c^
LSD for G*MAP				7.9^c^				10.4^c^				15.6^d^
SE for G				2.6				2.6				3.9
SE for MAP				1.5				1.2				3.3
SE for G*MAP				3.9				3.5				7.7

CV, Coefficient of variation; SE, Standard error; LSD, Least significant difference; G, Landraces (genotypes); MAP, Months after planting.

a Mean, mean over three harvest rounds.

b Mean, overall landrace site mean.

c *P* ≤ 0.001.

d *P* ≤ 0.01.

**Table 5:** Root starch content (g/kg) six cassava landraces evaluated in three sites at three different harvesting rounds (MAP)

	CHAMBEZI				AMANI				MAGADU			
Landraces (G)	9 MAP	12 MAP	15 MAP	Mean^a^	9 MAP	12 MAP	15 MAP	Mean^a^	9 MAP	12 MAP	15 MAP	Mean^a^
Kalolo	229.8	261.4	211.9	234.4	279.3	292.7	260.9	277.6	256.6	291.9	249.0	265.8
Kibandameno	316.3	329.0	300.0	315.1	319.4	334.9	300.4	318.2	315.2	348.2	291.1	318.2
Kilusungu	236.5	253.7	240.9	243.7	319.5	346.6	302.3	322.8	283.2	299.9	269.8	284.3
Kiroba	295.1	303.5	268.8	289.1	321.1	327.0	303.3	317.1	284.2	323.5	274.1	294.0
Msenene	262.1	286.3	243.4	263.9	278.7	294.2	237.6	270.2	288.0	297.4	255.7	280.4
Nyamkagile	276.2	291.6	275.3	281.4	337.4	346.6	302.8	328.9	322.9	334.6	311.8	323.1
Mean^b^	269.3	287.6	256.7	271.2	309.2	323.7	284.5	305.8	291.7	315.9	275.2	294.3
CV (%)		1.3				1.4				2.4		
LSD for G		0.41^c^				5.2^c^				6.6^c^		
LSD for MAP		0.24^c^				2.8^c^				5.1^c^		
LSD G*MAP		0.60^c^				7.2^c^				11.7^d^		
SE G		1.9				2.3				2.9		
SE MAP		1.2				1.4				2.5		
SED G*MAP		3.0				3.6				5.6		

CV, Coefficient of variation; SE, Standard error; LSD, Least significant difference; G, Landraces (genotypes); MAP, Months after planting.

a Mean, mean over three harvest rounds.

b Mean, overall landrace site mean.

c *P* ≤ 0.001.

d *P* ≤ 0.01.

**Table 6:** Starch yield (t ha^−1^) of six cassava landraces evaluated in three sites at three different harvesting rounds (MAP)

	CHAMBEZI				AMANI				MAGADU			
Landraces	9 MAP	12 MAP	15 MAP	Mean^a^	9 MAP	12 MAP	15 MAP	Mean^a^	9 MAP	12 MAP	15 MAP	Mean^a^
Kalolo	2.4	2.2	4.1	2.9	0.7	1.0	2.6	1.4	0.4	1.1	2.2	1.2
Kibandameno	2.4	1.7	3.3	2.4	1.8	1.0	7.4	3.4	0.6	2.8	2.9	2.1
Kilusungu	1.7	1.9	4.2	2.6	1.8	2.7	5.5	3.3	1.1	3.7	3.2	2.7
Kiroba	1.3	7.0	6.7	5.0	2.1	4.3	5.7	4.0	0.6	3.2	3.4	2.4
Msenene	1.7	3.6	9.6	5.0	2.5	4.3	3.3	3.3	0.4	2.4	1.7	1.5
Nyamkagile	2.1	2.5	5.3	3.3	1.7	2.5	3.5	2.3	1.6	1.7	2.9	5.3
Mean^b^	1.9	3.1	5.5	3.5	1.8	2.6	4.7	3.0	0.6	2.5	2.7	1.9
CV (%)				7.2				7.8				10.0
LSD for G				0.4^c^				0.23^c^				1.9^c^
LSD for MAP				0.3^c^				0.16^c^				0.13^c^
LSD G*MAP				0.7^c^				0.4^c^				1.02^c^
SE for G				1.3				0.1				0.09
SE for MAP				0.91				0.1				0.06
SE G*MAP			0.27				0.2					0.16

CV, Coefficient of variation; SE, Standard error; LSD, Least significant difference; G, Landraces (genotypes); MAP, Months after planting.

a Mean, mean over three harvest rounds.

b Mean, overall landrace site mean.

c *P* ≤ 0.001.

Variations in cyanide content (Table 7) were highly due to genotype and genotype by environment interaction (GEI) (*P *≤ 0.001). Nyamkagile had cyanide content below 30 ppm at Magadu. Furthermore, Kibandameno also had low cyanide content at Bagamoyo site at 9 and 12 MAP. The trend showed that each landrace gave different response at each harvesting time and it varied with location. Nyamkagile was the only landrace which showed decrease in cyanide content with age. However, the trend and cyanide concentration differed for each environment as demonstrated by significant GEI (*P* ≤ 0.001) (Table 7).

**Table 7:** Cyanide content (ppm) of six cassava landraces evaluated in three sites at three different harvesting rounds (MAP)

	CHAMBEZI				AMANI				MAGADU			
Landraces	9 MAP	12 MAP	15 MAP	Mean^b^	9 MAP	12 MAP	15 MAP	Mean^b^	9 MAP	12 MAP	15 MAP	Mean^b^
Kalolo	100	100	200	133.3	400	400	150	316.7	200	100	100	133.3
Kibandameno	30	100	50	60.0	100	100	100	100.0	50	100	75	75.0
Kilusungu	40	600	400	346.7	400	400	150	316.7	400	400	400	400.0
Kiroba	50	100	800	316.7	200	100	200	166.7	100	100	40	80.0
Msenene	50	75	100	75.0	100	50	40	63.3	100	100	100	100.0
Nyamkagile	125	100	100	108.3	200	100	75	125.0	30	15	25	23.3
Mean^c^	65.8	179.2	275	173.3	233.3	191.7	119.2	181.4	146.7	135.8	123.3	135.3
CV (%)				7.1				13.9				7
LSD for G				19.8^a^				30.6^a^				14.21^a^
LSD for MAP				10.9^a^				21.6^a^				8.39^a^
LSD G*MAP				26.7^a^				53.0^a^				20.01^a^
SE for G				5.4				11.24				5.5
SE for MAP				3.5				7.28				3.8
SE for G*MAP				8.9				18.4				9.5

CV, Coefficient of variation; SE, Standard error; LSD, Least significant difference; G, Landraces (genotype); MAP, Months after planting.

a *P* ≤ 0.001.

b Mean, mean over three harvest rounds.

c Mean, overall landrace site mean.

### Variation in traits in response to effect of genotype and environment

The analyses of variances (ANOVA) for fresh root yield, starch content, starch yield, and cyanide content across sites and harvesting rounds (Table 8) show significant differences for genotype and environment main effects as well as the GEI, except for root starch content where the genotype x round of harvesting was not significant. Furthermore, genotypes contributed most to the variability of starch (40.6%) and cyanide (38.8%) content than other sources of variation. Kiroba performed well in terms of fresh root yield at Amani and Chambezi, whereas Msenene had the highest fresh root yield at Chambezi and the lowest fresh root yield at Magadu. Nyamkagile yielded high at Chambezi site and low yield were observed at Magadu. Furthermore, Nyamkagile had the highest starch content at Amani and Magadu sites, while Kibandameno had the highest starch content at Chambezi. Nevertheless, Kalolo had the lowest starch content across all locations.

**Table 8:** ANOVA table for fresh root yield, starch yield, starch and cyanide content for six cassava landraces

		Fresh root yield (t ha^−1^)			Root starch content (g/kg)		
Source of variation	df	Sum of Squares (SS)	Contribution to SS (%)	Mean square	SS	Contribution to SS (%)	Mean square
Replicates	2	3.9		1.9	8.9		4.5
Location (L)	2	1261.2^a^	14.43	630.6	33,491.8^a^	19.26	16,745.9
Landrace (G)	5	652.8^a^	7.47	130.6	70,546.4^a^	40.56	14,109.3
Harvest (MAP)	2	3345.6^a^	38.29	1672.8	36,338^a^	20.89	18,169
G × MAP	10	438.6^a^	5.02	43.9	2069.4ns	1.19	206.9
G × L	10	698.3^a^	7.99	69.8	19,924.9^a^	11.46	19.92
G × L × MAP	34	2218.8^a^	25.39	65.3	6663.6^a^	3.83	196
Residual	106	119.3	1.36	1.1	4892		46.2
Total	171	8738.5			1,73,935		
		Starch yield (t ha^−1^)			Root cyanide content (ppm)		
Source of variation	df	SS	Contribution to SS (%)	Mean square	SS	Contribution to SS (%)	Mean square
Replicates	2	0.61		0.30	3.7		3.7
Location (L)	2	214.31^a^	31.90	107.16	43,672.2^a^	1.61	21,836
Landraces (G)	5	59.95^a^	8.92	11.99	10,51,211.1^a^	38.80	21,0242
Harvest (MAP)	2	77.46^a^	11.53	38.73	11,938.9^a^	0.44	5969.4
G × MAP	10	39.19^b^	5.83	3.92	3,28,983.3^a^	12.14	32,898
G × L	10	47.43^a^	7.06	4.74	3,69,450.0^a^	13.64	36,945
G × L × MAP	44	220.81^a^	32.87	5.02	8,88,444.4^a^	32.79	37,019
Total	181	671.73			27,09,400		

G, Landraces (genotypes); n, Not significant; L, Location; MAP, Months after planting; harvest round.

a *P* ≤ 0.001.

b *P* ≤ 0.01.

In terms of starch content, the trend per site was Amani > Magadu > Chambezi. For fresh root yield, Chambezi > Amani > Magadu (Table 8 and Fig. S1). Chambezi had sandy soils, which are suitable for cassava production because of easy soil penetration and expansion of the growing root. Amani had sand clay loamy soils, which are appropriate for water retention and thus provided a good distribution of soil water for long period after the start of dry season. Magadu had lowest fresh root yields, which might have been attributed to the clay soils and relatively lowest amount of rainfall received during the growing period. Kilusungu recorded high mean cyanide content across the environment, and Msenene had the lowest at Chambezi and Amani. Bagamoyo site exhibited increase in cyanide content, Amani site displayed a decrease trend, and Magadu displayed almost constant trend over the growth period (Table 8 and Fig. S1).

### Superiority and stability for starch yield and cyanide content for cassava landraces

The potential of cassava as a biomaterial for different industries depends on its yield and starch content, and consequently starch yield. Also, cassava use for food is constrained by its cyanide content. In this study, these two important traits have been found to be significantly influenced by GEI. Genotype by environment biplot was done to elucidate the stability of the landraces under different environments in terms of starch yield and cyanide content. The cultivar superiority measure encompasses calculations (across environments) of the mean square difference between the performance of a variety and the best variety within a given environment, measuring mean performance and stability simultaneously. GGE biplots enables visual comparison of the location, genotype, and their interaction.

**Figure 2:**
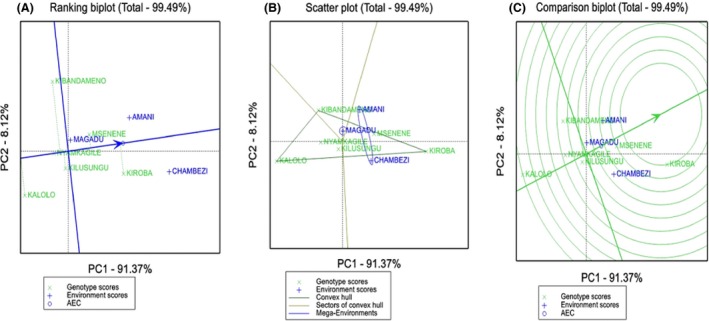
Genotype by Environment (GGE) biplots (A) showing ranking of cassava landraces based on mean cassava starch yield and stability performance across three environments (B) scatter plot for which‐won‐where (superiority) showing the best landrace for each environment (C) the average environment coordination (AEC) view to rank landraces relative to an ideal genotype (center of the concentric circle).

The GGE biplot analysis showed that PC1 and PC2 accounted for 91.4 and 8.12% (total = 99.49) variation, respectively, for starch yield (Fig. 2). The average environment coordinates (AEC) of the GGE biplot (Fig. 2A) shows mean starch yield and stability of the six landraces in three environments. A stable genotype will be very close to the AEC and will have a short perpendicular line. Therefore, Nyamkagile was stable but had below average starch yield (Fig. 2A), followed by Msenene with high starch yield but recorded low at Chambezi. Kiroba was superior and performed well at Chambezi and Amani site; meanwhile, Kalolo had the least across environments. In terms of test environment, Magadu had shortest vector from AEC, indicating stability and little discriminating information on genotype (least representative). Chambezi site had the longest vector indicating the highest discriminative and representative (Fig. S2).

The GGE biplot depict landrace which had the best performance at each location (which‐won‐where biplot). A convex hull with three vectors was formed with Kiroba, Nyamkagile, and Kalolo as the vertex cultivars (Fig. 1B). Kiroba was winning landrace in mega environment (Chambezi and Amani). No environment fell into sector with Kalolo as the vertex, indicating that it was not the best in any environment. Figure 1C illustrated ideal genotype (the center of concentric circles), which should have both high mean performance and high stability across environment. From the plot, Kiroba is closer to the ideal genotype followed by Msenene, indicative of high yielding and adapted genotypes.

**Figure 3:**
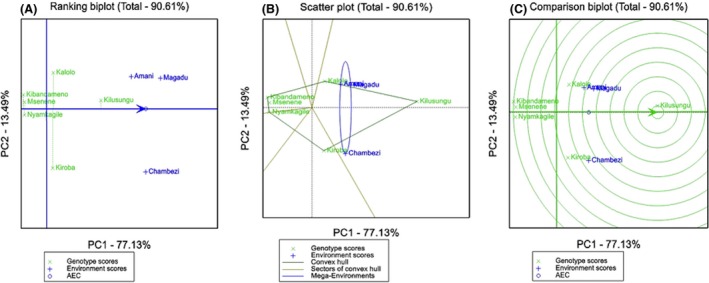
Genotype by Environment (GGE) biplots (A) showing ranking of cassava landraces based on mean cassava cyanide content and stability performance across three environments (B) scatter plot for which‐won‐where (superiority) showing the winning landrace for each environment (C) the average environment coordination (AEC) view to rank landraces relative to an ideal genotype.

The GGE biplot analysis for cyanide content showed that PC1 and PC2 accounted for 77.1% and 13.5%, respectively (Fig. 3A). The analysis showed that Kilusungu had the highest cyanide content across all environments (stable). Kiroba had highest cyanide content at Chambezi. Kibandameno, Msenene and Nyamkagile had the lowest cyanide content across environment. Figure 3B illustrate that both trial sites formed a mega environment, this implies that they both had comparable discriminative ability for cyanide content. This can also be explained by length of their vectors as shown in Figure 3A.

## Discussion

The results from this study showed significant differences between genotypes, locations, and rounds of harvesting for all the traits evaluated. However, significant one and two way interactions were also detected for all the traits. This implies that the genotypes responded differently to the locations and rounds of harvesting. This phenomenon was also the same as reported by Egesi et al. (2007) who observed similar results for cassava fresh root yield. Our findings also agree with those of Benesi et al. (2008) who reported that optimal harvest time was genotype and environment dependent.

Yield increase observed from 9 to 15 MAP, and a huge increase in fresh root yield was observed between 12 and 15 MAP, after commencement of rainfall period (March–May). The dry period that existed between January and March (9–12 MAP) caused low carbohydrate partitioning in the storage root (Santisopasri et al. 2001; El‐Sharkawy 2006). In contrary, dry matter and starch content increased up to 12 MAP and decreased at 15 MAP, a trend also observed by Sagrilo et al. (2006) except for Magadu site. Accumulation of dry matter increased, until physiological rest (12 MAP) and from that period onwards reserve carbohydrates are mobilized for synthesis of new vegetative growth (Sagrilo et al. 2006; Benesi et al. 2008). Cultivars recorded high starch content at Amani site; this can be explained by soils at Amani having high potassium content compared to the other sites and the fact that it received rain showers throughout the year. This has been also reported by Benesi et al. (2008).

Previous studies showed that cyanide content varied with genotypes and across environment (Bokanga et al. 1994; Burns et al. 2012). This study also found significant difference (*P *≤* *0.001) between landraces and GEI, therefore confirming previous studies. McMahon et al. (1995) and Bokanga et al. (1994) reported that cyanogenic potential of cassava is also age dependent and it decreases as root grows. Although trend was not the same for most landraces used in this study and varied across the environments except for Nyamkagile. This further demonstrated the genotype dependency of cyanide production (Bokanga et al. 1994). Furthermore, it has been reported that mechanism involving HCN metabolism is very complicated and involves several genes (Møller 2010). Amani site showed a decrease cyanide with age, as landraces did not exhibit considerable water stress (Cardoso et al. 2005). Chambezi site had a considerably increase in cyanide this could be explained by, a 5 months drought experienced during growing period. El‐Sharkawy (2006) and Burns et al. (2012) reported that during water stress cassava plant exhibits high cyanide content compared to the well‐watered cassava. Kiroba recorded high cyanide content (800 ppm) during 15 MAP at Bagamoyo site. The instability of Kiroba for cyanide content confirms reports from farmers in Kisarawe and Muheza districts who complained that it tasted bitter at certain times and places, and were therefore reluctant to adopt it because they grew cassava mainly for the fresh market. Cyanide value of 1090 and 1550 ppm have been reported by Mlingi and Bainbrigde (1994) for Tanzania bitter cassava cultivars.

Analysis by GGE biplot further elucidated the performance of landraces across trial sites (Yan and Tinker 2006; Akinwale et al. 2011) for cyanide content and starch yield. In terms of starch yield, Kiroba was superior at Chambezi and Amani, and Msenene was a superior landrace at Magadu site. Kilusungu had the highest cyanide across all environments but had average starch yield and which was stable across environments.

## Conclusion

Genotype and environment had a profound effect on all traits analyzed. These variations indicate significant genetic diversity present in farmer fields that can be trapped to increase yield potential at different locations. Genotypes should be selected for specific adaptation to environments. Furthermore, optimal harvesting time should also be based on specific variety and location for all the traits we have investigated in this study. The study has also confirmed effect of genotype and GEI on cyanide content of cassava roots. Kilusungu displayed high cyanide and average starch yield, and was stable across environments. Therefore, it can be recommended for starch production. The study has also showed the potential toxicity of Kilusungu. Consequently, as a safety measure, farmers should be emphasized on the need for proper processing prior to consumption.

However, from the GGE biplots, the most stable and above starch yielding landrace was Msenene. Kiroba was the winning landrace for starch yield at Amani and Chambezi. The most stable and safe landrace for fresh root consumption was Nyamkagile. For maximum starch yield, Kiroba should be harvested at 12 MAP and highest starch content for Kiroba was recorded at Amani, although in terms of starch yield it was higher at Chambezi. Amani and Bagamoyo formed a mega environment, thus shared same superior landraces for maximum starch yield.

## Conflict of Interest

None declared.

## Supporting information


**Figure S1.** Effect of time of harvesting (9, 12, 15 months after planting) on (A) fresh root yield (B) dry matter content (C) starch content (D) cyanide content, of six cassava landraces across three sites.
**Figure S2.** GGE Scatter plot showing discrimination and representativeness of the environments (sites) using the starch yield data of the six cassava landraces.
**Figure S3.** GGE Scatter plot showing discrimination and representativeness of the environments (sites) using the cyanide content of the six cassava landraces.
