# Supplementary material for: Effect of genotype and genotype by environment interaction on total cyanide content, fresh root, and starch yield in farmer‐preferred cassava landraces in Tanzania
Source: Food Sci Nutr. 2016 Feb 9;4(6):791–801. doi: 10.1002/fsn3.345 (PMC5090642; doi:10.1002/fsn3.345)
Supplement: Supplementary file 1 — Figure S1. Effect of time of harvesting (9, 12, 15 months after planting) on (A) fresh root yield (B) dry matter content (C) starch content (D) cyanide content, of six cassava landraces across three sites. Figure S2. GGE Scatter plot showing discrimination and representativeness of the environments (sites) using the starch yield data of the six cassava landraces. Figure S3. GGE Scatter plot showing discrimination and representativeness of the environments (sites) using the cyanide content of the six cassava landraces. [file FSN3-4-791-s001.docx]

**SUPPLEMENTAL DATA**

| 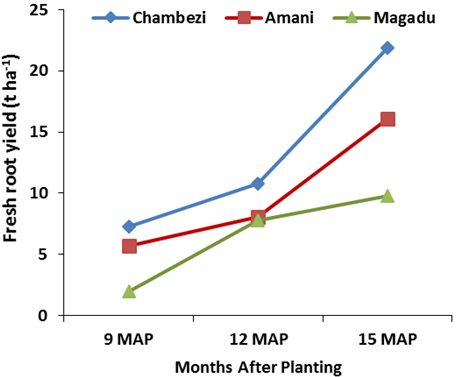  **A** | 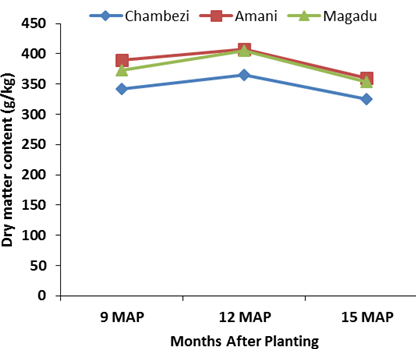  **B** | 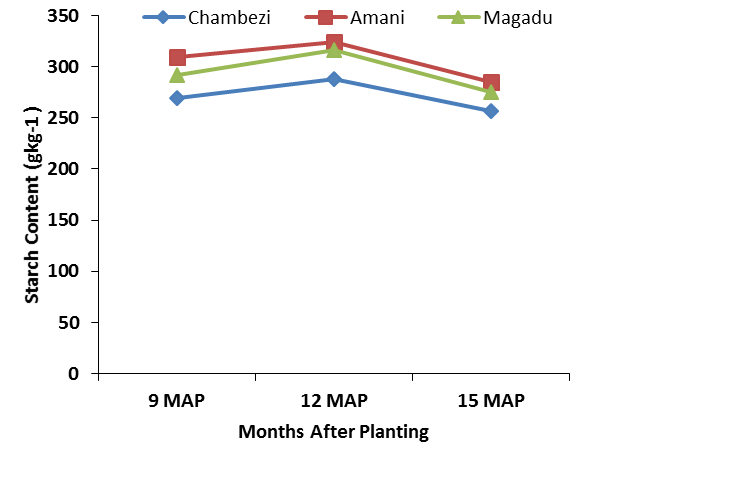  **D**  **C** | 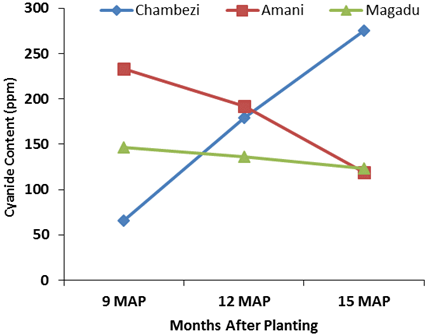 |
| --- | --- | --- | --- |

**Supplemental figure 1. Effect of time of harvesting (9, 12, 15 months after planting) on A) fresh root yield B) dry matter content C) starch content D) cyanide content, of six cassava landraces across three sites.**


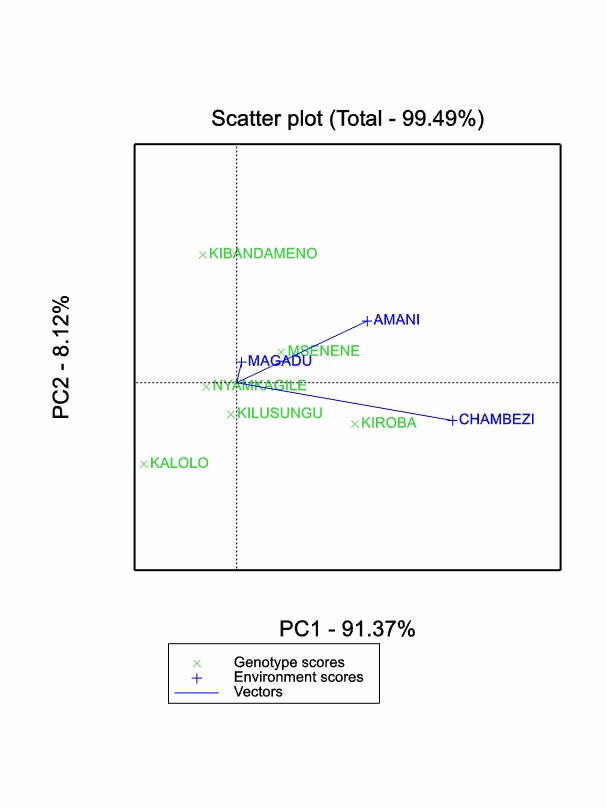


Supplemental figure 2: Supplemental figure2. GGE Scatter plot showing discrimination and representativeness of the environments (sites) using the starch yield data of the six cassava landraces.


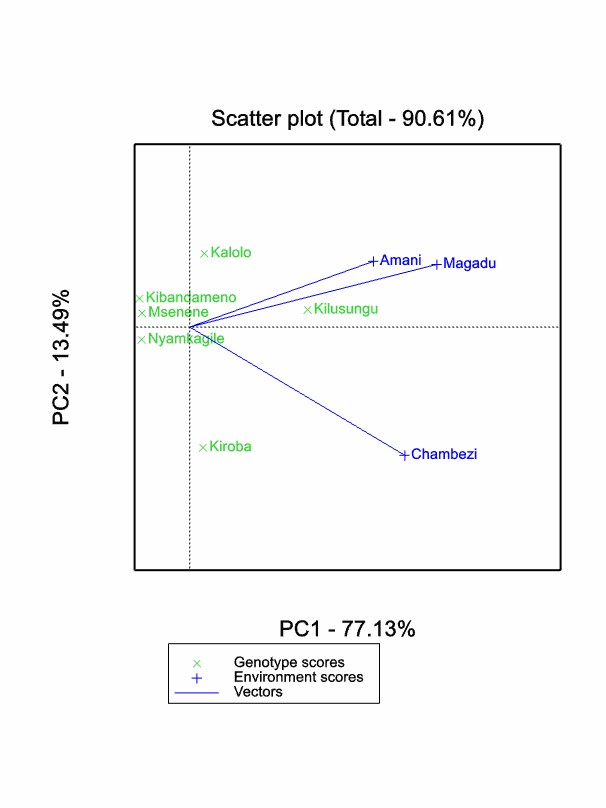


Supplemental figure 3. GGE Scatter plot showing discrimination and representativeness of the environments (sites) using the cyanide content of the six cassava landraces.
